# Ovarian Cancer in the Older Manitoban Population—Treatment Tolerance and Cancer-Related Outcomes: A Manitoba Ovarian Cancer Outcomes (MOCO) Group Study

**DOI:** 10.3390/curroncol31030102

**Published:** 2024-03-05

**Authors:** Lesley F. Roberts, Pascal Lambert, Mark W. Nachtigal, Alon D. Altman, Erin Dean

**Affiliations:** 1 Department of Obstetrics, Gynecology and Reproductive Sciences, University of Manitoba, Winnipeg, MB R3E 0L8, Canada; mark.nachtigal@umanitoba.ca (M.W.N.); aaltman@cancercare.mb.ca (A.D.A.); edean@cancercare.mb.ca (E.D.); 2 Department of Epidemiology and Cancer Registry, CancerCare Manitoba, Winnipeg, MB R3E 0V9, Canada; plambert@cancercare.mb.ca; 3 Paul Albrechtsen Research Institute, CancerCare Manitoba, Winnipeg, MB R3E 0V9, Canada; 4 Department of Biochemistry and Medical Genetics, University of Manitoba, Winnipeg, MB R3E 0J9, Canada

**Keywords:** geriatric oncology, ovarian cancer, treatment tolerance

## Abstract

Background: In Canada, individuals with gynecologic reproductive organs (ovaries, fallopian tubes, uterus) over the age of 70 comprise a large proportion of epithelial ovarian cancer patients. These patients often have co-morbidities, polypharmacy, or decreased functional status that may impact treatment initiation and tolerance. Despite this, there is limited evidence to guide treatment for older patients diagnosed with ovarian epithelial carcinoma. Methods: This is a retrospective study with data from Manitoba, Canada. The data were obtained from the Manitoba Ovarian Cancer Database, the Manitoba Cancer Registry, and electronic health records. All individuals with epithelial ovarian, fallopian tube, or peritoneal cancer diagnosed between 2009 and 2018 were identified. Patients aged > 70 at the time of diagnosis were included in the study cohort. Results: Four hundred and forty individuals were included. The majority had advanced stage disease (56%). Moreover, 59% of patients received no chemotherapy. Of the patients who received chemotherapy, 20% received <2 cycles and 21% required a dose reduction due to toxicity. Univariable and multivariable analysis identified advanced stage (*p* < 0.001), treatment modality (*p* < 0.001), and advanced age at diagnosis (*p* < 0.001) with poorer overall survival. Conclusions: Our study demonstrated a high rate of chemotherapy dose reduction and discontinuation in the elderly epithelial ovarian cancer population. Further research is needed to identify risk factors for treatment discontinuation and intolerance in this population.

## 1. Introduction

Epithelial ovarian cancer encompasses five major histotypes, high-grade and low-grade serous, clear cell, endometrioid and mucinous and is the most lethal form of gynecologic cancer [1]. In Canada, an estimated 3100 individuals were newly diagnosed with epithelial ovarian cancer in 2023, with an estimated 1950 individuals dying from the disease [1]. Thus, the estimated 5-year overall survival for individuals with epithelial ovarian cancer in Canada is 44% [1], with Manitoba having the highest survival rate in the country [2]. The risk of developing epithelial ovarian cancer increases with age, until reaching a plateau of 0.4% per decade starting at age 70 [1]. While the median age at diagnosis in the United States is 63, individuals over the age of 65 account for 45% of diagnoses and 65% of deaths [3]. This trend is made more concerning by evidence from several retrospective studies identifying poorer outcomes in patients diagnosed at later age [2,4]. As the population ages and life expectancy increases, the number of individuals over 65 being diagnosed and dying from epithelial ovarian cancer is expected to rise. Therefore, it is imperative to identify the factors that contribute to mortality in the elderly epithelial ovarian cancer population. Epithelial ovarian cancer is typically treated as one disease, but as our understanding of this diverse group of diseases evolves, the development of more personalized care based on disease histotype and patient age will likely need to change in order to develop clinical management plans to improve outcomes.

There is limited evidence surrounding the best treatment for older patients diagnosed with epithelial ovarian cancer and the reasons for poorer outcomes are not fully understood. Epithelial ovarian cancer patients are typically treated with a combination of chemotherapy [six–nine cycles of a platinum agent (carboplatin) and a taxane (paclitaxel)] either before (neoadjuvant) or after (adjuvant) surgical debulking. Further lines of chemotherapy that this patient population may receive include repeated use of the platinum/taxane combination, pegylated liposomal doxorubicin, gemcitabine, or topotecan. Geriatric patients are often excluded from randomized clinical trials, and therefore the best course of treatment for this patent population is poorly understood [5,6]. Individuals over the age of 70 comprise a large proportion of epithelial ovarian cancer patients and often have co-morbidities, polypharmacy, or decreased functional status [7,8]. Consequently, the evidence base for the treatment of epithelial ovarian cancer is drawn primarily from experiences with younger, healthier patients. Older patients are often treated similarly to their younger counterparts, but there is an emerging body of primarily retrospective literature focusing on treatments and outcomes of elderly patients with epithelial ovarian cancer. Research shows that older patients have higher complication rates and lower success rates associated with aggressive cytoreductive surgery [9], and thus trends in care are shifting towards primary chemotherapy alone for elderly patients [10,11].

The primary objective of this study is to develop a comprehensive understanding of how older patients (i.e., patients aged ≥ 70 at the time of diagnosis) with epithelial ovarian cancer tolerate and respond to different treatment paradigms.

## 2. Materials and Methods

This retrospective cohort study was approved by the University of Manitoba Research Ethics Board (REB #HS22929) and by CancerCare Manitoba. Funding was provided by the University of Manitoba Department of Obstetrics, Gynecology and Reproductive Sciences Resident Research Fund.

Data (demographic, diagnostic, treatment, and survival) were obtained from the Manitoba Cancer Registry and the Manitoba Ovarian Cancer Database associated with the Manitoba Ovarian Biobank Program, housed within the Manitoba Tumor Bank at CancerCare Manitoba. Additional clinical information and individual medical histories were extracted by a chart review from the CancerCare Manitoba patients’ charts. The cohort database contained anonymized personal health identification numbers, age, diagnosis, histologic features, stage, surgical procedures performed, adjuvant or neoadjuvant treatments, number of recurrences, recurrent treatments, and overall survival status. Information such as dose reductions and dose interruption, as proxy measures for treatment toxicity, were collected from the patient chart review.

All individuals identified by ICD-10 and ICD-O-3 codes, indicating a cancer of the ovary including the fallopian tube, adnexa, and primary peritoneum, in the Manitoba Ovarian Cancer Database between the years 2009 and 2018 were identified and validated by the chart review. Patients that were diagnosed based on cytology only were considered unclassified epithelial ovarian cancer, as a histologic classification was not available. Additionally, patients who were diagnosed based on radiologic and biochemical findings alone, without histopathologic diagnosis, were labelled as unclassified epithelial ovarian cancer. Only patients aged ≥ 70 at the time of diagnosis were included in the study cohort. Patients were excluded if pathology was not confirmed via the chart review. Missing data elements from the databases were collected retrospectively from the electronic medical health record.

Age ranges were broken down into cohorts by decade (i.e., 70–79, 80–89, ≥90) (Table 1), and baseline characteristics were reviewed (histology, stage, and location). Treatment types and outcomes were analyzed by age cohort (Table 2). Descriptive statistics for the number of chemotherapy cycles and dose reduction were calculated. Cox regression analyses were performed to examine models predicting overall survival. Treatment variables were included as time-varying predictors. A *p*-value of 0.05 was used as the threshold for statistical significance. Likelihood ratio testing was used for selecting the inclusion of predictors in the analysis of the 70 to 79 age subgroup. The same predictors were included in the 80 to 89 age subgroup analysis for consistency, but we also used likelihood ratio testing to consider additional predictor variables. Univariable and multivariable analyses were performed.

## 3. Results

A total of 440 individuals with gynecologic reproductive organs (ovaries, fallopian tubes, uterus), 70 years of age and older, who were diagnosed with epithelial ovarian cancer between 2009 and 2018 were identified. The mean age of diagnosis was 78.4. The majority of patients had serous or unclassified epithelial ovarian cancer (Table 1). Twenty-six patients had low grade tumors and 120 had high grade tumors. Using FIGO 2014 staging, 36% had stage III disease and 20% had stage IV disease; however, a more granular evaluation indicated that staging differed depending on the age group in this cohort of patients. For 24% of patients, the stage was not documented and was therefore classified as unknown. Interestingly, the 90+ age group exhibited the highest percent of unknown/undocumented stage (12/27 patients; 44.4%). On individualized chart reviews, it was clear that the majority of these patients had advanced disease (stage III, IV, or unknown accounting for 80% of these patients).

The incidence and type of treatment (no treatment; chemotherapy alone; surgery alone; neoadjuvant chemotherapy plus surgery) was analyzed by age cohort (Table 2). A patient was classified as having received chemotherapy if they received at least one dose of systemic intravenous chemotherapy. While there was similar incidence of treatment intervention in each decade, the majority of patients received no treatment (70–79: 46%, 80–89: 67.8%). In the total cohort (i.e., all patients > 70), 13.2% received chemotherapy only, 18% underwent debulking only, 12.3% had neoadjuvant chemotherapy, followed by surgery and adjuvant chemotherapy. First-line chemotherapy (neoadjuvant/adjuvant) was platinum-based in 98% of patients (Appendix A). Interestingly, only two patients (<0.05%) underwent the classic standard care for epithelial ovarian cancer, i.e., primary debulking surgery followed by adjuvant chemotherapy. Patients over the age of 90 were not included in Table 2 as only three patients (11%) received any treatment at all. The utilization of PARP inhibitors was not available to patients in Manitoba outside of the clinical trial (which excluded patients over the age of 70) during the study time frame and therefore were not included as a treatment modality.

Over half of the patients (59%) received no chemotherapy at any point in their disease course. Of those who received any chemotherapy, 14% received one line of treatment, 11% received two lines, and 7% received three lines (Table 3). Only 8% of patients received more than three lines of chemotherapy. Among the patients who received chemotherapy, 22.7% received ≤two cycles of the prescribed line of chemotherapy. In addition to this high rate of non-completion, we noted that 21.1% of all patients had a chemotherapy dose reduction; specifically, dose reduction occurred in 15.3% of patient aged 70–79 and 37.0% of patients aged 80–89. Dose interruption also occurred in 13.2% of all patients with 8.2% in patients aged 70–79 and 25.9% in patients aged 80–89. Unfortunately, due to the low number of patients over 90 years, we were unable to report the rates of dose reduction or interruption.

**Figure 1:**
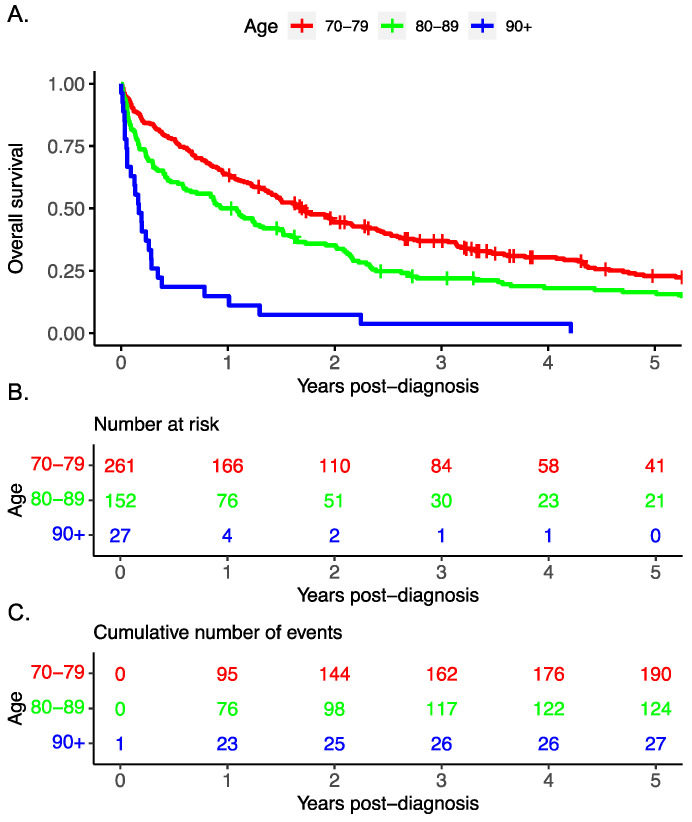
Overall survival of patient cohort shown by (**A**) decade, (**B**) risk score with (**C**) corresponding number of events.

The overall survival decreased with advancing age (Figure 1). Cox regression multivariable analysis identified advanced stage, treatment modality, and advanced age to be associated with worse survival.

Our analyses indicate that for individuals 70 to 79, treatment modality is significantly related to survival, with surgery alone having the best survival (Table 4). In addition, advanced age and advanced stage are related to worse survival. For those aged 80 to 89, treatment modality is significantly related to survival, with surgery alone having the best survival, while advanced stage is associated with worse survival. No treatment or chemotherapy alone are associated with poorer survival. The analysis of combined therapy (i.e., surgery and chemotherapy combined) was limited by small numbers. A univariable analysis was performed for the assessment of treatment, age, histology, stage, residence and income. Significant differences were noted in age, stage, histology, and treatment (Appendix B). These differences remained on subsequent multivariable analyses. A multivariable survival analysis for treatment effects was performed using surgery alone as the reference group, as this group had the best survival outcome. The entire treatment variable of the model was significant (*p* = 0.006); however, there was no significant difference between surgery only and surgery combined with chemotherapy with respect to overall survival (HR 1.35, CI 0.79–2.30). Conversely, chemotherapy alone and no treatment had significantly worse outcomes than surgery alone (Table 4).

## 4. Discussion

There is a growing need to understand how older epithelial ovarian cancer patients respond to different treatment modalities to ensure this population is not being undertreated. While the standard of care for treatment of epithelial ovarian cancer at the time of our study consisted of debulking surgery plus six cycles of platinum-based chemotherapy, only 41% of patients in our cohort received chemotherapy, of which a further 22.7% received no more than two cycles. Only 12.7% (56) of patients received a combination of debulking surgery and chemotherapy, whereas only 8% of our patient population received more than three lines of chemotherapy. We noted that the non-treatment rate increased with age. These data suggest that our older patients are either not being offered chemotherapy, not wanting chemotherapy or are unable to complete chemotherapy that has been started. Using dose reduction or dose interruption as a proxy measure, we interpret our results to infer that a high number of patients tolerated treatment poorly due to adverse effects, highlighting the difficulty of treating patients in this age group. If we also examine the rate of “unknown” stage or unclear pathology (i.e., unclassified carcinoma or other), these results further reinforce that elderly patients do not receive the same or equivalent workup and investigations as a younger cohort, i.e., smaller samples likely collected from guided biopsies and staging based on imaging versus full staging procedures. Once again, this leads to a question of patient ability to undergo investigations and treatment and questions patient willingness for investigations and interventions. This appears to be consistent with previous studies showing similar results of elderly patients being treated less aggressively [10].

The low numbers of patients receiving standard of care therapy and the high percentage of dose reduction or early dose interruption is in contrast with younger epithelial ovarian cancer patients. Treatment efficacy rates and survival percentages are modeled off clinical trials comprised primarily of patients under the age of 75, with high treatment completion rates and low–moderate dose reduction rates [12]. This emphasizes that patient selection for treatment is clinically very challenging and is an area where improvement and ongoing research are required. Additionally, it makes it clear that standard clinical trial data cannot easily be extrapolated to the older epithelial ovarian cancer population.

In 2018, Schuurman et al. published a large retrospective population-based study of individuals aged 70 years and older diagnosed with advanced stage epithelial ovarian cancer in the Netherlands [10]. They noted that these elderly patients were more often less aggressively treated. While the standard of care consists of aggressive cytoreductive surgery and multi-agent chemotherapy, the treatment of these patients often shifted to primary chemotherapy instead of surgery. A recent randomized controlled trial investigated whether primary chemotherapy alone was appropriate for the older patient population. They compared standard carboplatin and paclitaxel doublet chemotherapy versus single agent carboplatin in patients 70 years or older. The trial was closed early due to the comparatively poor survival rates in patients receiving single agent carboplatin [11]. Treatment guided by age alone may result in under-treatment of this subgroup of patients who otherwise would be sufficiently fit to pursue standard of care treatment; this is highlighted by our results where 59% of patients over 70 did not receive chemotherapy and 22.7% of patients who started on chemotherapy only completed ≤two cycles. For those older patients who are treated with the standard of care for epithelial ovarian cancer (i.e., surgery and chemotherapy), some may appear functionally well but have unrecognized underlying frailty that causes significant clinical and functional decompensation following aggressive treatment [13]. Currently, there are no well-validated predictors of candidacy for aggressive standard of care for advanced epithelial ovarian cancer in the geriatric population.

Our data are consistent with previous retrospective studies indicating that outcomes and tolerance to treatment are poorer in the elderly population. However, this conflicts with prospective, randomized controlled trials demonstrating that treating older patients with modified, less aggressive regimens results in worse outcomes [11]. This is likely due to a selection bias within the trial, favoring the enrollment of healthier, more functional geriatric epithelial ovarian cancer patients, i.e., patients who could receive standard course treatment.

Surgery alone as a treatment modality had the best overall survival, in contrast to no treatment or chemotherapy alone, which were associated with the poorest survival. By contrast, published work indicated that aggressive cytoreductive surgery was associated with higher complication rates and lower success rates [9]. Combined treatment (i.e., surgery and chemotherapy) did not demonstrate a significantly improved overall survival on multivariate analyses (HR 1.35; CI 0.79–2.30; *p* = 0.006). These results are unexpected, given that the gold standard treatment of epithelial ovarian cancer is a combination of surgery and chemotherapy. However, our analysis is limited by low numbers, as the proportion of patients in our cohort who received combination treatment was smaller than expected (12.7%). Additionally, these results are impacted by the inclusion of epithelial ovarian cancer histologies more likely to present at an earlier stage and thus have a better prognosis, such as mucinous or endometrioid histology. For these patients, surgery alone may be sufficient for early stage disease due to excellent prognosis; surgery may also have been offered more often in isolated disease and quickly refused in advanced stage cancers. Unfortunately, analysis based on histology was not possible due to small patient numbers.

Fundamentally, these conflicting bodies of evidence demonstrate the clinical challenge in assessing fitness and making decisions for treatment intervention in the geriatric population. The discrepancy between numerical age and functional age can be challenging to identify clinically but has significant impacts on treatment tolerance. Multiple assessments for clinical frailty indexes, comprehensive geriatric assessments, and even biomarker assessments of age have been investigated [14]. The development of more robust criteria for fitness for therapy within surgical and oncology patients may help alleviate this conflict by directing care and avoiding either under- or over-treatment of geriatric patients.

As a retrospective analysis, our results are primarily limited by significant clinical and patient selection bias; specific decisions made by patients, families and clinicians cannot be determined from the medical record. Additionally, we were limited to the data coded into the database or recorded into the electronic medical record, which was not always complete. Moreover, our statistical analysis was limited in our oldest cohort (>90), due to small numbers. The inclusion of “unknown” stage data introduces uncertainty, but given the small number of records, particularly in the older age groups, we do not feel this unknown data can be excluded from the study. We have included a sensitivity analysis in Appendix D, in which the unknown data have been excluded. As predicted, with removal of these data, the numbers are very small which limits the reliability of these results. As this is a retrospective study, we are looking at real-world evidence and the reality of practice, which often includes significant unknowns. We therefore believe that it is important to keep these data in our primary analyses.

Our study is one of the first examining the treatment rates and outcomes in the elderly population with epithelial ovarian cancer. Our rates of treatment in the 80–89 cohort appear lower than previously reported, and this is the first study that attempted to look at survival in patients over the age of 90. Overall, our results suggest that elderly patients are treated less aggressively than their younger cohorts, but the reasoning and decision-making processes cannot be determined from this study. Further prospective research is required into the management of epithelial ovarian cancer in geriatric patients since generalizations from standard trials cannot be used for the elderly population. Further qualitative research may also help contribute to our understanding of decision making in our elderly population, helping guide clinicians in their counselling and patient support.

## Figures and Tables

**Table 1:** Demographics of patient population overall and broken down by age range.

		70–79	80–89	90+
Patient Population		261	152	27
Histology	Serous	114 (43.7) *	48 (31.6)	3 (11.1)
	Endometrioid	10 (3.8)	3 (2.0)	0
	Clear cell	7 (2.7)	2 (1.3)	0
	Mucinous	8 (3.1)	7 (4.6)	2 (7.4)
	Unclassified epithelial	90 (34.5)	74 (48.7)	19 (70.4)
	Other	32 (12.3)	18 (11.8)	3 (11.1)
FIGO stage	I	24 (9.2)	22 (13.4)	3 (11.1)
	II	27 (10.3)	11 (7.2)	1 (3.7)
	III	107 (41.0)	44 (28.9)	7 (25.9)
	IV	49 (18.8)	34 (22.4)	4 (14.8)
	Unknown	54 (20.7)	41 (27.0)	12 (44.4)
Location	Urban	170 (65.1)	95 (62.5)	16 (59.3)
	Rural	91 (34.9)	57 (37.5)	11 (40.7)

* Values presented as number (% of total).

**Table 2:** Treatment by age cohort (decade).

Treatment	70–79		80–89	
	N ^1^	%	N	%
Chemotherapy ^2^ + surgery	47	18	9	5.9
Surgery only	56	21.5	22	14.5
Chemotherapy only	38	14.6	18	11.8
No treatment	120	46	103	67.8

^1^ N = number; ^2^ this includes neoadjuvant and/or adjuvant chemotherapy.

**Table 3:** Number of chemotherapy cycles and lines achieved by the patient cohort.

	Line Number					
	1		2		3	
Number of Cycles ^2^	N ^1^	%	N	%	N	%
1	16	14.5	10	10.8	5	6.0
2	9	8.2	13	14.0	5	6.0
3	56	50.9	16	17.2	13	15.7
4–6	18	16.3	47	50.5	50	60.2
>6	11	10.0	7	7.5	10	12.0

^1^ N = number. ^2^ This includes neoadjuvant and adjuvant chemotherapy.

**Table 4:** Multivariable survival analysis.

		70–79				80–89			
		HR	95% CI	*p*	Type III *p*	HR	95% CI	*p*	Type III *p*
Treatment	Surgery alone	Reference				Reference			
	No treatment	1.93	1.24–3.00	0.004	0.006	3.76	1.92–7.36	<0.001	0.001
	Chemotherapy only	2.26	1.30–3.93	0.004		3.01	1.34–6.76	0.008	
	Chemotherapy + surgery	1.35	0.79–2.30	0.260		1.75	0.64–4.75	0.272	
Age at diagnosis	Per 10 years	1.60	0.98–2.62	0.061	0.061	0.98	0.50–1.91	0.950	0.061
Stage	Stage I–II	Reference				Reference			
	Unknown	3.29	1.98–5.47	<0.001	<0.001	3.48	1.93–6.27	<0.001	<0.001
	III–IV	2.36	1.52–3.67	<0.001		3.04	1.8–5.16	<0.001	

HR = hazard ratio; CI = confidence interval.

## Data Availability

The data presented in this study is available in this article.

## Appendix A

**Table A1:** First-line chemotherapy regimen for patients in the cohort receiving systemic chemotherapy (neoadjuvant or adjuvant).

Chemotherapy Regimen	N
None	327
Carboplatin (AUC5)/paclitaxel q21 days	71
Carboplatin (AUC5)/paclitaxel q28 days	38
Single agent carboplatin (AUC5)	1
Carboplatin (AUC5)/dose dense paclitaxel q21 days	1
Single agent paclitaxel q21 days	1

## Appendix B

**Table A2:** Univariable and multivariable analysis for predicting overall survival. Further explanation of the multivariable + interaction term model is provided in Appendix C.

		Univariable				Multivariable				Multivariable + Interaction			
		HR	95% CI	*p*	Type III *p*	HR	95% CI	*p*	Type III *P*	HR	95% CI	*p*	Type III *p*
Treatment	Surgery Alone	Reference				Reference				Reference			
	No treatment	3.23	2.28–4.59	<0.001	<0.001	2.09	1.44–3.02	<0.001	<0.001	0.02	0.00–3.11	0.133	<0.021
	Chemo only	3.70	2.42–5.66	<0.001		1.61	1.02–2.57	0.043		10.15	0.02–6213.76	0.479	
	Chemo and surgery	2.40	1.55–3.71	<0.001		1.39	0.87–2.23	0.163		5.06	0.00–5951.44	0.653	
Age at diagnosis	Per 10 years	1.74	1.46–2.07	<0.001	<0.001	1.70	1.41–2.04	<0.001	<0.001	1.17	0.65–2.10	0.610	0.610
Histology	Mucinous	Reference				Reference				Reference			
	Unclassified	3.35	1.90–5.90	<0.001	<0.001	2.33	1.28–4.25	0.006	<0.001	2.60	1.43–4.74	0.002	<0.001
	Serous carcinoma	1.18	0.66–2.09	0.573		1.12	0.61–2.05	0.717		1.24	0.68–2.28	0.482	
	Clear cell, endometrioid	0.99	0.47–2.07	0.971		1.39	0.65–2.97	0.399		1.55	0.72–3.34	0.260	
	Other	1.64	0.88–3.06	0.120		1.63	0.86–3.10	0.135		1.82	0.95–3.46	0.070	
Stage	I–II	Reference				Reference				Reference			
	Unknown	3.79	2.67–5.39	<0.001	<0.001	2.65	1.82–3.86	<0.001	<0.001	2.59	1.78–3.78	<0.001	<0.001
	III–IV	2.73	2.01–3.70	<0.001		2.43	1.71–3.45	<0.001		2.47	1.74–3.50	<0.001	
Residence	Rural	Reference											
	Winnipeg/Brandon	0.95	0.77–1.17	0.634	0.634								
Income	R4–R5	Reference											
	R1–R3	1.27	0.88–1.83	0.195	0.195								
	U4–U5	Reference											
	U1–U3	1.18	0.89–1.56	0.249	0.249								
Treatment	Surgery alone									Reference			
x age interaction	No treatment									1.77	0.95–3.32	0.074	0.010
	Chemo only									0.80	0.35–1.82	0.588	
	Chemo and surgery									0.83	0.33–2.11	0.701	

HR = hazard ratio; CI = confidence interval; Winnipeg/Brandon = major urban centers; R1–R5 = rural; U1–U5 = urban.

## Appendix C

**Table A3:** Specific analysis of multivariate analysis + interaction term for treatment and age, holding all other predictors in the model at their average values. Hazard ratios are provided for different treatments at the specific ages indicated. This demonstrates that chemotherapy alone and chemotherapy + surgery compared to surgery alone have effects that decrease as age increases, while no treatment relative to surgery alone has an effect that increases as age increases.

Treatment	Age	HR	95% CI	*p*
Chemotherapy only versus surgery alone	70	2.05	0.95–4.45	0.069
	80	1.63	0.99–2.71	0.058
	90	1.30	0.42–4.03	0.649
Chemotherapy and surgery versus surgery alone	70	1.42	0.68–2.96	0.351
	80	1.18	0.65–2.15	0.579
	90	0.99	0.25–3.90	0.985
No treatment versus surgery alone	70	1.31	0.73–2.38	0.366
	80	2.33	1.55–3.51	<0.001
	90	4.13	1.72–9.93	0.002

HR = hazard ratio; CI = confidence interval.

## Appendix D

**Table A4:** Sensitivity analysis for multivariate survival analysis (Table 4) removing all patients with unknown stage.

		70–79				80–89			
		HR	95% CI	*p*	Type III *P*	HR	95% CI	*p*	Type III *P*
Treatment	Surgery alone	Reference				Reference			
	No treatment	1.74	1.07–2.84	0.027	0.030	2.97	1.49–5.91	0.002	0.013
	Chemotherapy only	2.34	1.26–4.33	0.007		2.32	0.97–5.52	0.058	
	Chemotherapy + surgery	1.38	0.77–2.47	0.283		1.58	0.55–4.54	0.399	
Age at diagnosis	Per 10 years	1.49	0.86–2.61	0.158	0.158	1.41	0.60–3.29	0.434	0.434
Stage	I–II	Reference				Reference			
	III–IV	2.30	1.46–3.61	<0.001	<0.001	3.03	1.77–5.16	<0.001	<0.001

HR = hazard ratio; CI = confidence interval.
